# Acidified Acetonitrile Extraction and Ultra-High Performance Liquid Chromatography–Tandem Mass Spectrometry for Multiresidue Determination of 136 Pesticides in Tahiti Lime (*Citrus latifolia*)

**DOI:** 10.3390/foods15172967

**Published:** 2026-08-24

**Authors:** Karolaine da Silva Albarnaz, Juliana Diniz de Barros Kuntz, Luana Floriano, Franciele Fátima Machado, Martha Bohrer Adaime, Renato Zanella, Osmar Damian Prestes

**Affiliations:** Laboratory of Pesticide Residue Analysis (LARP), Department of Chemistry, Federal University of Santa Maria, Av. Roraima, 1000, Santa Maria 97105-900, RS, Brazil

**Keywords:** food safety, citrus fruits, acidic pesticides, multiresidue analysis, analytical method validation

## Abstract

The determination of pesticide residues in citrus fruits remains analytically challenging due to their high acidity and complex chemical compositions, which can compromise extraction efficiency and promote significant matrix effects, particularly for acidic compounds. In this study, an acidified acetonitrile-based QuEChERS method was developed and validated for the multiresidue determination of 136 pesticides in Tahiti lime (*Citrus latifolia*) using ultra-high-performance liquid chromatography coupled with tandem mass spectrometry (UHPLC–MS/MS). The proposed approach employs 1% formic acid in acetonitrile and omits the conventional clean-up step, aiming to improve analyte recovery and analytical throughput. Method validation was performed according to SANTE guidelines, demonstrating satisfactory linearity (R^2^ ≥ 0.99 for all analytes), limits of quantification of 0.01 mg kg^−1^, and recoveries within 70–120%, with relative standard deviations ≤ 20% for the vast majority of compounds. Notably, the method achieved consistent and reliable performance for 12 acidic pesticides, which are typically problematic in conventional QuEChERS approaches. Application to 22 real samples revealed the presence of 14 pesticide residues, with some compounds exceeding European Union (EU) maximum residue limits. The proposed method provides a simple, efficient, and robust alternative for comprehensive pesticide monitoring in citrus fruits, with particular advantages for the determination of acidic compounds in highly complex matrices.

## 1. Introduction

Citrus fruits, including lemon, orange, tangerine, and lime, are among the most widely consumed commodities worldwide, making citrus production a highly relevant agricultural sector from both economic and food safety perspectives [1]. In Brazil, citrus cultivation is particularly significant, with lemon production reaching approximately 1.5 million tons in recent years and expanding across multiple regions, including the southeast, northeast, and south [2]. This large-scale production is accompanied by intensive pesticide use, as citrus crops are highly susceptible to pests and diseases, requiring frequent applications of insecticides and acaricides to maintain yield and fruit quality [3].

Despite their agronomic importance, the widespread use of pesticides raises significant concerns regarding food safety and consumer exposure [4]. To mitigate these risks, maximum residue limits (MRLs) have been established by regulatory agencies such as European Food Safety Authority, Brazilian Health Regulatory Agency (ANVISA), etc. [5,6]. Consequently, the development of reliable, sensitive, and robust analytical methods for monitoring pesticide residues in citrus fruits is essential to ensure compliance with regulatory standards and to protect public health [7].

From an analytical perspective, citrus fruits represent one of the most challenging matrices for pesticide residue determination [8]. Tahiti lime (*Citrus latifolia*), in particular, is characterized by high acidity (pH 2–3) and a complex chemical composition comprising thousands of compounds, including organic acids, flavonoids, and essential oils [9,10]. These characteristics contribute to pronounced matrix effects (ME) in mass spectrometry and can significantly affect extraction efficiency, especially for pH-sensitive compounds such as acidic pesticides [11]. Conventional multiresidue methods often struggle to achieve adequate recoveries for these analytes due to pH-dependent ionization and interactions with sorbents during clean-up steps [12].

The QuEChERS (Quick, Easy, Cheap, Effective, Rugged, and Safe) method has become the most widely used sample preparation approach for pesticide residue analysis in food matrices, owing to its simplicity, versatility, and compatibility with both gas and liquid chromatography [13,14,15]. Several analytical methods have been reported for the determination of pesticide residues in citrus fruits using QuEChERS combined with LC–MS/MS [12,16,17]. Most studies employed citrate-buffered QuEChERS for multiresidue analysis [18,19], whereas a smaller number of investigations in lemon matrices adopted acetate-buffered or acidified QuEChERS procedures [20]. Although these methods generally demonstrated satisfactory performance for conventional pesticides, they included a relatively limited set of acidic pesticides and did not specifically address the influence of extraction pH on their recoveries. Moreover, most approaches relied on conventional clean-up steps, which may promote losses of acidic analytes through interactions with PSA sorbents [21,22]. Consequently, there is still a need for simplified extraction strategies that can simultaneously determine conventional and acidic pesticides in highly acidic citrus matrices.

In parallel, advances in instrumental analysis, particularly ultra-high performance liquid chromatography coupled with tandem mass spectrometry (UHPLC–MS/MS), have enabled the simultaneous determination of hundreds of pesticide residues with high sensitivity, selectivity, and throughput [23,24,25].

In this context, the development of simplified and robust extraction strategies capable of efficiently recovering both conventional and acidic pesticides remains a critical challenge. Therefore, this study aimed to develop and validate an acidified acetonitrile-based QuEChERS method for the multiresidue determination of 136 pesticides in Tahiti lime (*Citrus latifolia*) using UHPLC–MS/MS. Particular emphasis was placed on evaluating the behavior of twelve acidic pesticides (2,4,5-T; 2,4,6-TCP; 2,4-D; bromacil; fluroxypyr; imazamox; imazapic; imazapyr; imazaquin; imazethapyr; MCPA and triclopyr) under modified extraction conditions, as well as assessing the feasibility of eliminating the clean-up step to improve analytical throughput while maintaining method performance. The applicability of the proposed method was further demonstrated through the analysis of commercial samples, providing relevant data for pesticide monitoring in citrus fruits.

## 2. Materials and Methods

### 2.1. Reagents, Analytical Standards and Materials

Solvents including acetonitrile, acetic acid, acetone, hexane, sodium hydroxide, and sodium chloride were purchased from Merck (Darmstadt, Germany). Methanol, ammonium formate, magnesium sulfate, sodium citrate dihydrate, and sodium hydrogen citrate sesquihydrate were obtained from Sigma-Aldrich (St. Louis, MO, USA). Ultrapure water was produced using a Milli-Q Direct UV3^®^ system (Millipore, Molsheim, France). Anhydrous sodium acetate was obtained from J.T. Baker (Phillipsburg, NJ, USA), and PSA sorbent was purchased from Supelco (Bellefonte, PA, USA). All analytical standards used in this work had a purity of at least 90% and were obtained from Dr. Ehrenstorfer (Augsburg, Germany), Sigma-Aldrich (St. Louis, MO, USA), and ChemService (West Chester, PA, USA). Atrazine-d5 was used as a surrogate standard to monitor the extraction procedure, whereas triphenyl phosphate was used as an internal standard to monitor instrument performance and stability during the analyses. Individual stock solutions of each analyte were prepared in acetonitrile or methanol at a concentration of 1000 mg L^−1^, taking into account the purity of the solid standards. A mixed standard solution at 10 mg L^−1^ was prepared by appropriate dilution of the stock solutions. In addition, two routinely used mixed standard solutions at 5 mg L^−1^ and 10 mg L^−1^ were prepared for method development and validation. All stock and working standard solutions were stored in amber glass bottles at temperatures below −10 °C.

### 2.2. Instrumentation and UHPLC–MS/MS Analysis

The general equipment used in this work included precision analytical balances (models AUX220 and UX220H, Shimadzu, Kyoto, Japan), a refrigerated centrifuge (NT825, Nova Técnica, Piracicaba, Brazil), a digital pH meter (8 × 15 MI, Firstlab, Pinhais, Brazil), a vortex mixer (VX-38, IonLab, Araucária, Brazil), an ultrasonic bath (Sonorex RK510, Bandelin, Berlin, Germany), and automatic micropipettes with variable volume (Brand, Wertheim, Germany). For chromatographic analyses, a UHPLC–MS/MS system (Xevo TQ XS, Waters Technologies, Milford, MA, USA), equipped with an Acquity binary pump UHPLC and triple quadrupole mass analyzer, was used. Data acquisition was performed using the MassLynx 4.1 software. Chromatographic separations were carried out on an Acquity UPLC^®^ HSS T3 column (100 mm × 2.1 mm i.d., 1.8 µm particle size). The mobile phase consisted of (A) water containing 5% (*v*/*v*) acetonitrile and (B) acetonitrile, both adjusted to 0.01% (*v*/*v*) acetic acid. The injection volume was 5 µL, the column temperature was maintained at 45 °C, and nitrogen was used as desolvation gas at a flow rate of 1000 L h^−1^. For the determination of pesticide residues, the percentage of organic phase (B) changed as follows: 0 min, 1%; 3.50 min, 40%; 12.50 min, 85%; 12.60 min, 99%; 15.10 min, 99%; 15.10 min, 1%; and 19.00 min, 1%. The flow rate was 0.500 mL min^−1^, and the total analysis time was 19 min. Table S1 shows the selected precursor and product ions and the corresponding collision energies used in the MS/MS multiple reaction monitoring (MRM) mode for both positive (ESI+) and negative (ESI−) electrospray ionization. Using MassLynx software, the most abundant transition (1st transition) was selected for quantification, and the second most abundant transition was used for identification.

### 2.3. Commercial Tahiti Lime (Citrus latifolia) Samples

Twenty-two commercial Tahiti lime samples were purchased from various supermarkets across Rio Grande do Sul, Brazil. Each sample corresponded to an individual commercial batch and was assigned a unique identification code (A1–A22). Approximately 1 kg of fruit was collected from each batch to ensure sample representativeness. Each commercial sample was assigned a unique identification code (A1–A22), corresponding to an individual batch collected from different supermarket. These sample codes are used throughout the manuscript, including in Table 1, for sample identification and traceability. Analytical blank samples consisted of Tahiti lime (*Citrus latifolia*) sourced from an organic production property where no pesticide applications were performed, confirmed free of target residues by UHPLC–MS/MS analysis. Fruits were processed whole (pulp, peel, seeds) according to *Codex Alimentarius* guidelines and stored in polypropylene containers at ≤−10 °C until analysis.

### 2.4. Sample Preparation (Acidified Acetonitrile Extraction)

In total, 10.0 ± 0.1 g of previous homogenized samples was weighed into 50 mL polypropylene centrifuge tubes. Extraction was performed by adding 10 mL acetonitrile containing 1% formic acid (*v*/*v*), followed by manual vortex agitation for 1 min. Salting-out extraction was achieved by sequential addition of 4 g anhydrous magnesium sulfate (MgSO_4_) and 1 g sodium chloride (NaCl), with immediate manual agitation for 1 min to prevent salt agglomeration. Tubes were centrifuged at 6000 rpm (1925× *g*) for 8 min at 10 °C. An aliquot of 0.2 mL supernatant (acetonitrile extract) was transferred to a 2 mL polypropylene conical tube with 0.8 mL ultrapure water (5-fold dilution). The diluted extract was filtered through PVDF syringe filters (13 mm × 0.2 μm) directly into UHPLC vials for immediate analysis. No additional cleanup (dSPE) was performed to preserve method simplicity and throughput.

### 2.5. Method Validation

Method validation was performed to ensure the quality criteria required according to the SANTE validation guide [26]. The acidified acetonitrile-based QuEChERS method for pesticide residue determination in Tahiti lime (*Citrus latifolia*) was validated by evaluating parameters such as selectivity, linearity, ME, limits of detection (LODs) and quantification (LOQs), accuracy through the recovery assay, precision (repeatability) and intermediate precision. Linearity was evaluated using calibration curves at concentrations of 0.50; 1.00; 2.00; 5.00; 10.00 and 20.00 µg L^−1^ prepared in acetonitrile (*n* = 3) and in matrix blank extract (*n* = 3). The ME was estimated by comparing the slope of the analytical curve prepared in the matrix with the curve prepared in the solvent (acetonitrile). The difference in the slopes of the curves prepared in the matrix and in the solvent was divided by the slope of the curve in the solvent and is expressed as a percentage of the ME. When the ME was greater than ±20%, it was considered significant [26]. Accuracy was evaluated through recovery assays of blank samples fortified with analyte mixtures at 0.01; 0.025 and 0.05 mg kg^−1^ (*n* = 6). The precision of the method was evaluated by means of the RSD (%), which was considered acceptable at ≤20%. Intermediate precision was evaluated by performing the analytical procedure on different days and injecting the calibration curve and the blank sample fortified at concentrations of 0.01; 0.025 and 0.050 mg kg^−1^ (*n* = 6). The method LOQ was defined as the lowest concentration of the spiked sample that showed recovery results between 70 and 120%, with an RSD ≤ 20%, according to the SANTE guidelines [26]. The LOD was estimated by dividing the LOQ by 3.33.

## 3. Results

### 3.1. Evaluation of UHPLC–MS/MS Analysis

**Figure 1:**
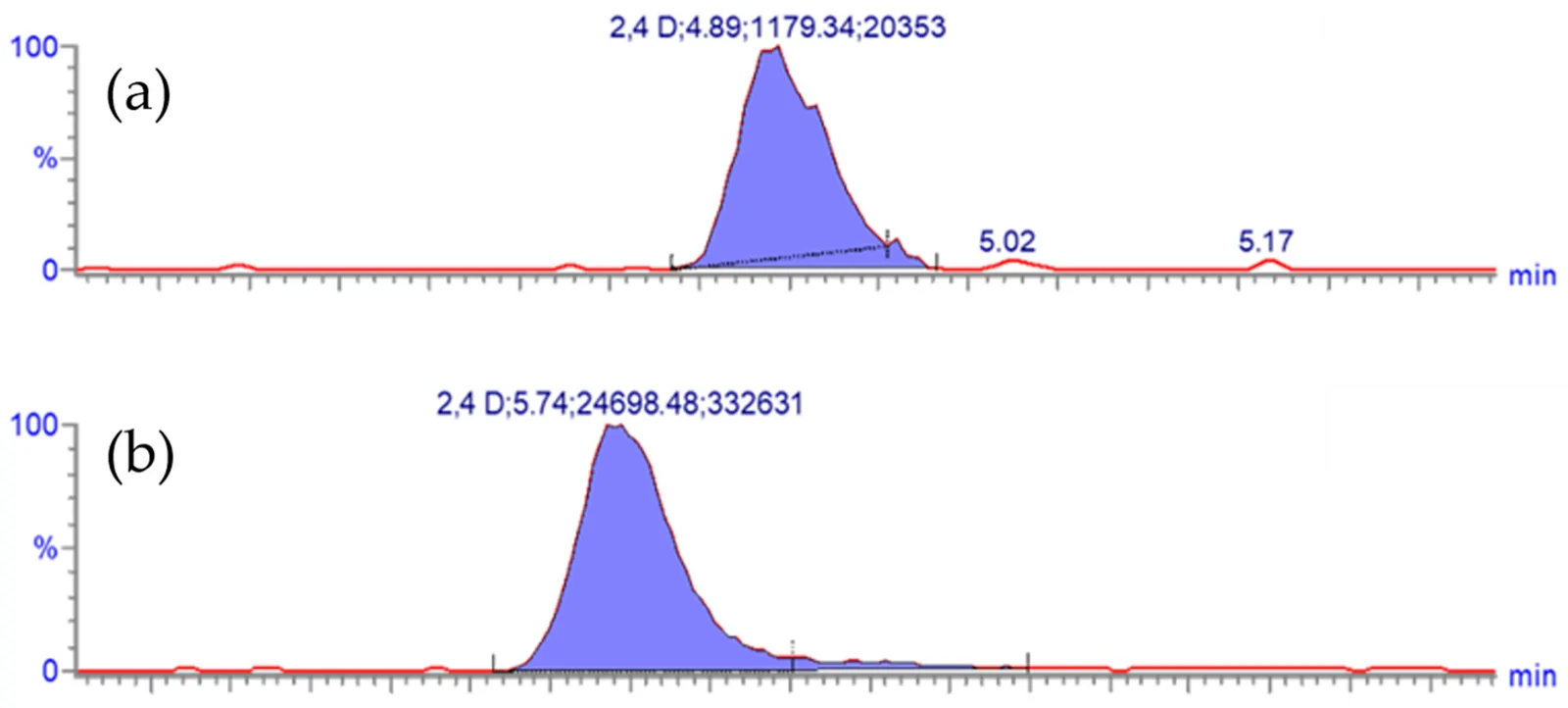
UHPLC–MS/MS MRM chromatograms of 2,4-D (0.5 µg L^−1^) comparing two mobile phase combinations evaluated during method optimization (**a**) Combination I, consisting of (A) water and (B) methanol/acetonitrile (1:1, *v*/*v*), both containing 0.1% formic acid (*v*/*v*) and 5 mmol L^−1^ ammonium formate; (**b**) Combination II, consisting of (A) water containing 5% acetonitrile (*v*/*v*) and (B) acetonitrile, both containing 0.01% acetic acid (*v*/*v*). The optimized mobile phase (**b**) provided improved peak symmetry and signal intensity.

Two mobile phase combinations were initially tested to optimize chromatographic performance, focusing on peak shape and signal intensity. Mobile phase combination I consisted of (A) water and (B) methanol/acetonitrile (1:1, *v*/*v*), both containing 0.1% formic acid (*v*/*v*) and 5 mmol L^−1^ ammonium formate. Combination II comprised (A) water with 5% acetonitrile (*v*/*v*) and (B) acetonitrile, both with 0.01% acetic acid (*v*/*v*). Both systems maintained at 0.225 mL min^−1^. Combination II (Figure 1) demonstrated adequate performance, exhibiting pronounced improvement in chromatographic peak shapes. This system provided more symmetrical peaks and higher signal-to-noise ratios (S/N improvement 25–180% for acidic pesticides), particularly for phenoxy acids and sulfonylureas.

This mobile phase was selected, as it aligns with EURL-SRM protocols for acidic pesticide analysis [27] and enabled robust quantification of twelve acidic pesticides alongside 136 routine multiresidue compounds. Optimized UHPLC–MS/MS parameters including cone voltages, collision energies, and MRM transitions for both quantification and identification, are detailed in Table S1.

### 3.2. Sample Preparation

**Figure 2:**
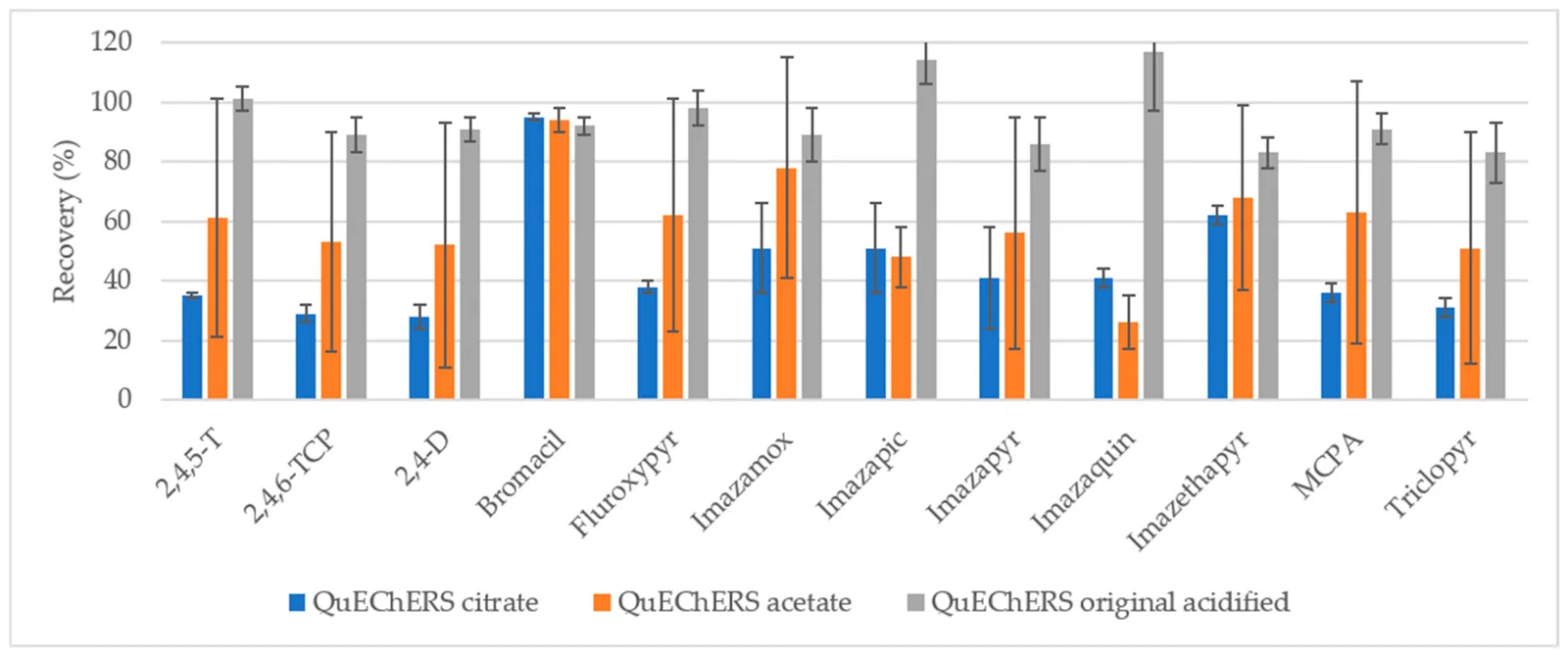
Comparative recoveries (%) of 12 acidic pesticides in *Citrus latifolia* obtained using citrate, acetate, and acidified original QuEChERS extraction methods. Error bars represent the relative standard deviation (RSD%, *n* = 3).

The literature review confirmed citrate QuEChERS as the predominant approach for citrus multiresidue analysis [28,29], while lemon-specific studies utilized acetate and original acidified variants [30,31]. Notably, while pesticide residue studies in Tahiti lime exist, none comprehensively evaluated all twelve acidic pesticides targeted here. Acidic pesticides warrant inclusion in multiresidue methods due to their extensive agricultural applications, for instance 2,4-D, among the most widely used phenoxy acid herbicide in Brazil, is authorized for over 17 crops including citrus, soybean, corn, and sugarcane, with commercialization exceeding 60,000 tons in 2024 [32,33]. The phenoxy acid compounds contain carboxyl groups (pKa 2.7–4.8) challenging conventional QuEChERS protocols through pH-dependent ionization and PSA adsorption losses, necessitating specialized acidified extraction strategies like the 1% formic acid acetonitrile method (pH 3.0–4.0) proposed here to ensure accurate quantification across diverse physicochemical properties while maintaining regulatory compliance [34]. Comparative evaluation of extraction methods (Figure 2) clearly demonstrates the inadequacy of acetate and citrate QuEChERS for acid herbicides in Tahiti lime (*Citrus latifolia*) both methods resulted in lower recoveries for most acidic pesticides, with several compounds failing to meet the SANTE acceptance criterion (recovery 70–120%). In addition, larger error bars observed for some analytes indicate greater variability in the extraction process. The proposed acidified method achieved superior, compliant recoveries (83–119% across all targets), confirming acidity, around pH 3.0–4.0; preventing carboxylate anion formation; and enabling quantitative partitioning into the organic phase. Although original, acetate, and citrate QuEChERS versions produce satisfactory results for conventional pesticides, this work presents a method with more acidic pH conditions to enable determination of acidic substances, aligning with EURL-SRM recommendations for non-ionized lipophilic partitioning into acetonitrile phase [27].

### 3.3. Method Validation

The validation parameters for the proposed multiresidue method demonstrate excellent analytical performance across 136 pesticides in Tahiti lime (*Citrus latifolia*) matrix. A summary of the validation results is presented in Table 1, while the complete validation data for each analyte are provided in Supplementary Table S2.

**Figure 3:**
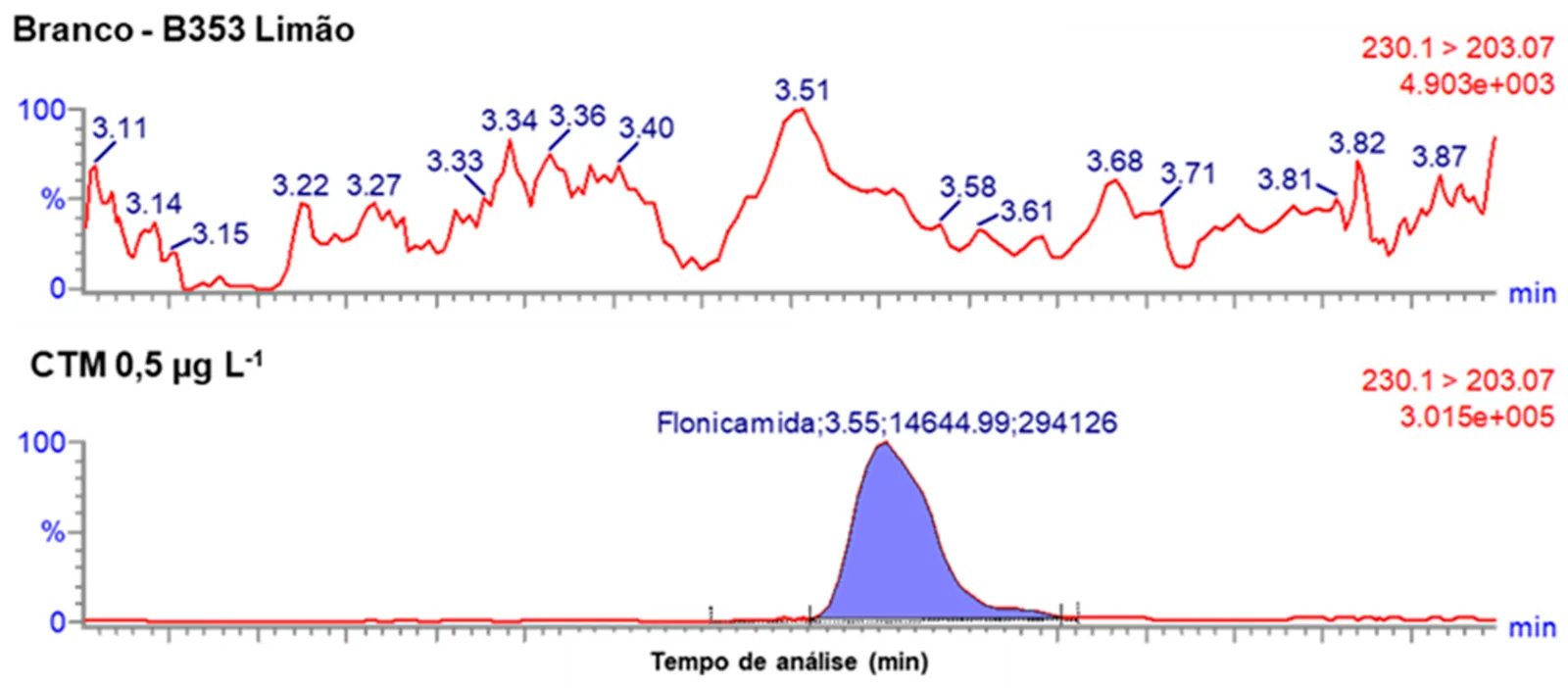
UHPLC–MS/MS chromatogram comparison demonstrating method selectivity for flonicamid analysis in Tahiti lime (*Citrus latifolia*): blank matrix extract versus 0.5 µg L^−1^ standard solution.

The method showed excellent selectivity. As example, flonicamid analysis in the sample extracts by UHPLC–MS/MS is shown in Figure 3, where the blank matrix extract completely lacks interfering peaks at the quantifier transition (230 > 203 *m*/*z*) when compared against a 0.5 µg L^−1^ standard solution. No endogenous matrix components coeluted with flonicamid above the method LOD. This clean chromatographic profile was observed for all 136 target analyzed pesticides, enabling matrix-matched calibration and meeting the EU SANTE/11312/2021 selectivity criteria for regulatory multiresidue monitoring [26].

In accordance with the data in Table S2, all the pesticides exhibited excellent linearity across the calibration range, with coefficient of determination (R^2^) ranging from 0.99 to 1.0000. This performance meets the SANTE/11312/2021 guidelines, which recommend R^2^ ≥ 0.98 for multiresidue methods [26]. The consistently high R^2^ values confirm the robust detector response and minimal baseline drift in the UHPLC–MS/MS system.

**Figure 4:**
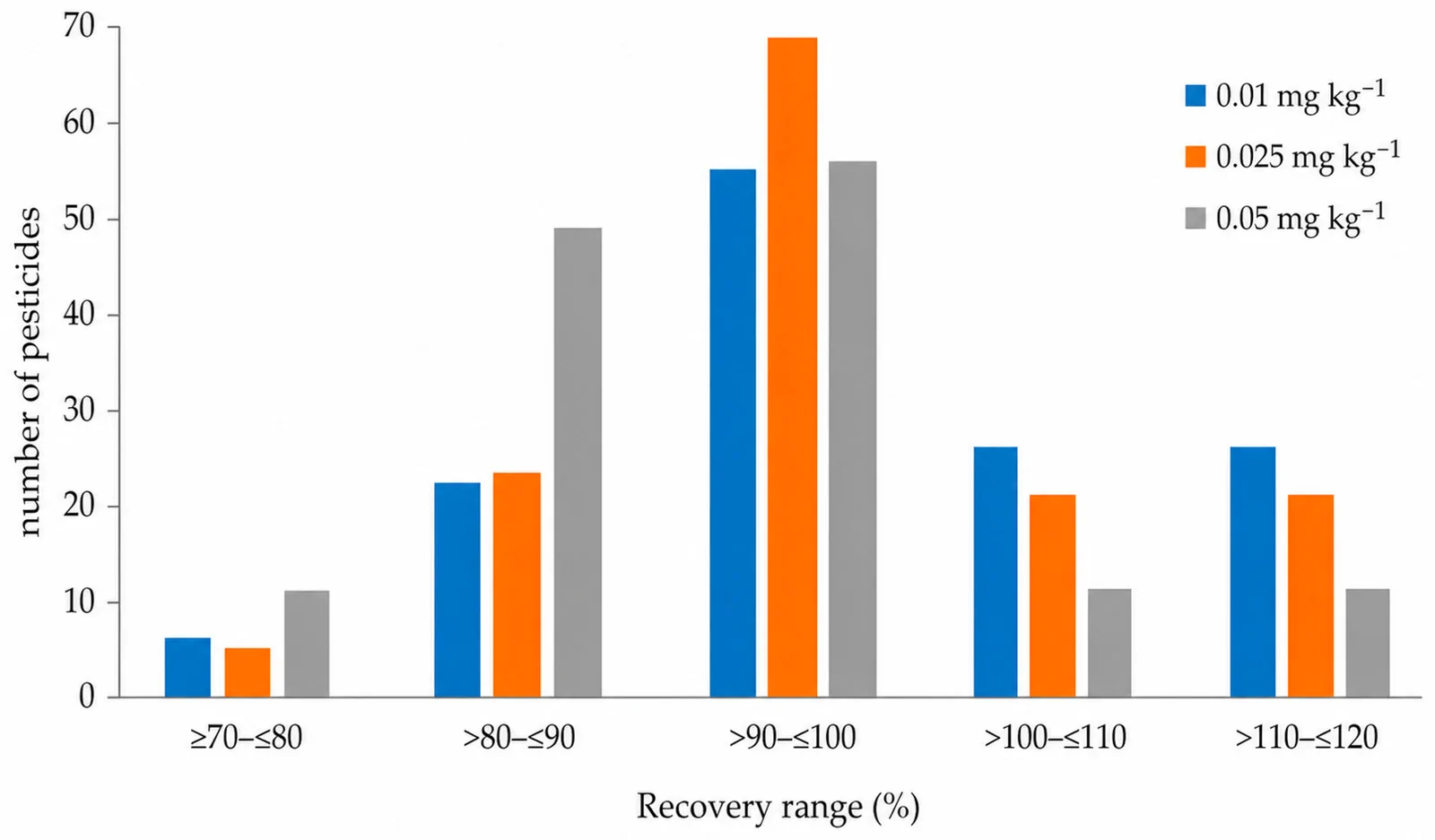
Distribution of recovery values for 136 pesticides at three fortification levels (0.01, 0.025 and 0.05 mg kg^−1^) in Tahiti lime (*Citrus latifolia*). Bars represent the number of analytes within each recovery interval.

The distribution of ME across 136 pesticides in Tahiti lime (*Citrus latifolia*) extracts revealed 48.5% negligible ME (±20%, 66 analytes), 48.5% considerable suppression (<−20%, e.g., 2,4-D −113%, azamethiphos −135%), and 3% enhancement (>20%, e.g., triclopyr +22%, chlorthiophos +52%). This variability stems from coelution of citrus-specific limonoids (limonin, nomilin) and flavonoids (naringin, hesperidin) competing for ESI charge (suppression) or stabilizing precursor ions (enhancement), as documented before in UHPLC–MS/MS multiresidue methods. Considering the pronounced matrix complexity observed, method validation was performed using matrix-matched calibration to compensate for signal suppression and enhancement, ensuring reliable quantification and compliance with regulatory requirements. In addition, atrazine-d5 was used as a surrogate standard to verify the consistency of the sample preparation procedure, while triphenyl phosphate was employed as an instrumental quality control standard to assess signal stability throughout the analytical sequence. The combined use of these quality assurance strategies ensured robust and reliable routine quantification despite the pronounced ME observed for several analytes. Trueness, evaluated as recovery% and RSD% at three fortification levels (0.01, 0.025, and 0.05 mg kg^−1^; *n* = 6), consistently fell within the 70–120% (RSD ≤ 20%) acceptance range (Table S2), as stipulated by the SANTE/11312/2021 guidelines [26]. Intermediate precision trials (different days/analysts) maintained adequate recoveries%, with RSDs generally ≤15%. The consistency between the intra- and inter-day (Figure 4) data for all the analytes indicates the robustness of the method against operator and temporal variability (e.g., imazalil: intra 87–95%, inter 87–94%). High RSDs (>15%) occurred in ~10% of the cases, often at low spikes (e.g., triclopyr).

**Figure 5:**
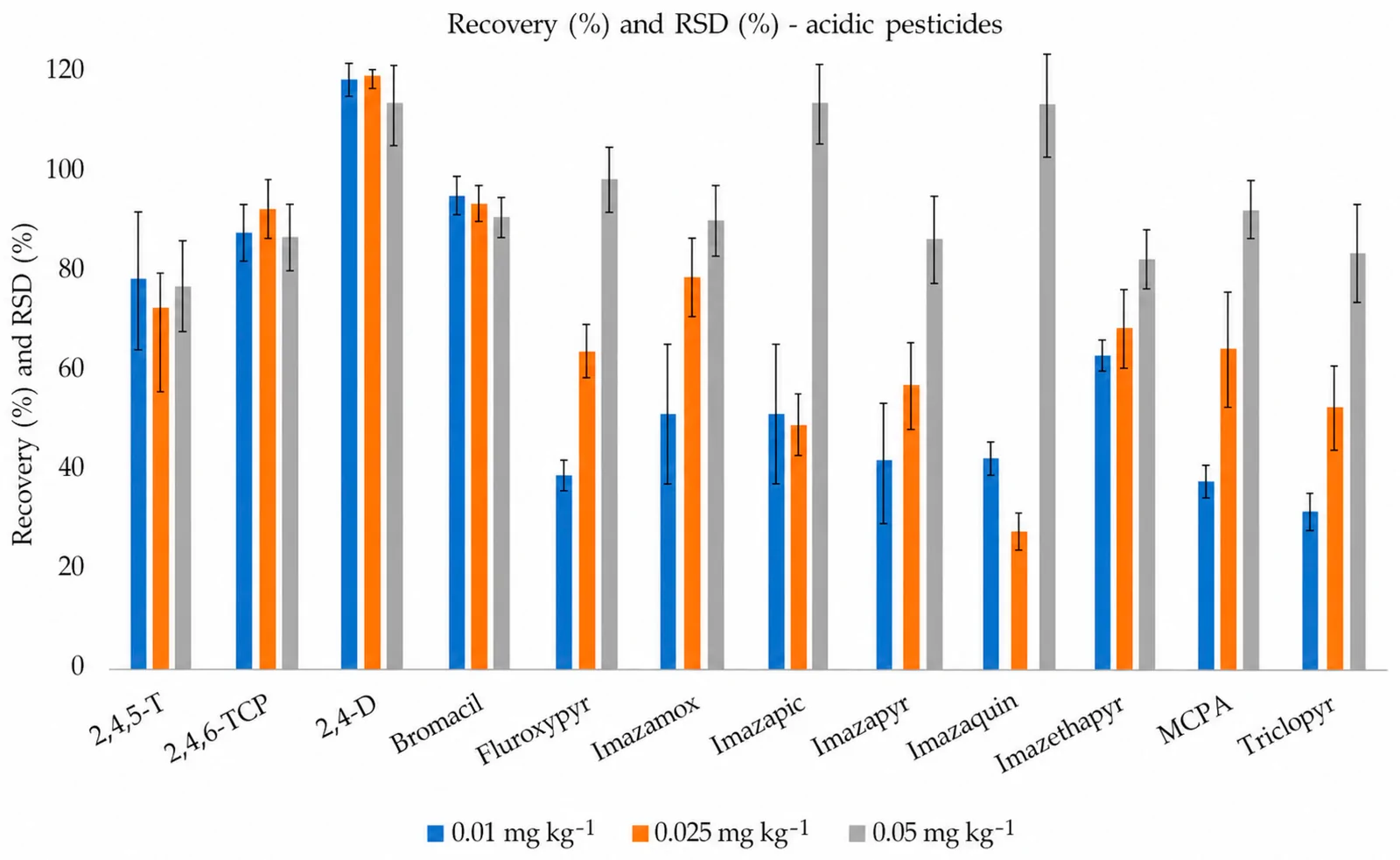
Recovery (%) and precision (RSD%, intraday; *n* = 6, error bars) of acidic pesticides in Tahiti lime (*Citrus latifolia*) at fortification levels of 0.01, 0.025, and 0.05 mg kg^−1^, determined using acidified QuEChERS and UHPLC–MS/MS.

The recovery results for acidic pesticides (Figure 5) in Tahiti lime (*Citrus latifolia*) demonstrate exceptional method performance considering the well-documented analytical challenges associated with these compounds (pKa 2.6–4.9) in conventional QuEChERS protocols.

All twelve acidic analytes (2,4,5-T; 2,4,6-TCP; 2,4-D; bromacil; fluroxypyr; imazamox; imazapic; imazapyr; imazaquin; imazethapyr; MCPA and triclopyr) achieved trueness and intermediate precision within regulatory 70–120% limits across fortification levels (0.01–0.05 mg kg^−1^, *n* = 6), with outstanding precision (RSD ≤ 18%): 2,4,6-TCP excelled (76–92%, RSD ≤ 5%), and imidazolinones proved robust (imazamox/imazapic/imazapyr/imazaquin/imazethapyr: 71–110%, RSD ≤ 13%), and even problematic phenoxy acids such as 2,4-D (82–119%, RSD ≤ 8%) and fluroxypyr (73–119%, RSD ≤ 9%) stabilized during inter-day trials despite characteristic low-level over-recoveries. Triclopyr showed 93–113% recoveries with RSD ≤ 18%, outperforming the literature reports where acidic pesticide recoveries in citrus samples frequently fall to 60–130% with an RSD > 20% because of pH-mediated partitioning losses in unbuffered or buffered extractions [31]. The acidified QuEChERS (1% formic acid) sample preparation used in this study successfully addressed these limitations, achieving regulatory compliance for all compounds typically requiring pH adjustment or extensive cleanup optimization. Other pesticides also exhibited adequate performance (Table S2), including triazines (e.g., atrazine: 77–104% recovery, RSD ≤ 17%), strobilurin fungicides (e.g., azoxystrobin: 89–103%, RSD ≤ 8%), neonicotinoids (e.g., acetamiprid: 92–116%, RSD ≤ 16%), and organophosphates (e.g., malathion: 72–100%, RSD 12–20%). This study provides a comprehensive validation of 136 pesticides in Tahiti lime (Citrus latifolia), including twelve acidic pesticides, thereby expanding the analytical scope for multiresidue monitoring in this complex citrus matrix. Imidazolinones (imazamox: 87–108%, RSD < 12%; imazapic: 91–105%, RSD ≤ 10%; imazethapyr: 89–110%, RSD < 11%) and sulfonylureas (chlorimuron-ethyl: 71–99%, RSD ≤ 12%; ethoxysulfuron: 75–102%, RSD ≤ 11%), critical for graminicide control in citrus orchards, are less commonly included in multiresidue methods for citrus matrices [35]. Similarly, cyazofamid (87–97%, RSD 2–10%) and vamidothion (95–120%, RSD ≤ 15%), addressed critical gaps in post-harvest fungicide/insecticide surveillance, where cyazofamid showed ME of 75–105% recovery in mandarin.

The method developed in this study was compared with other multiresidue sample preparation approaches previously reported for citrus matrices, including magnetic solid-phase extraction and different QuEChERS variants (Table 2).

When the main parameters are considered together, the acidified QuEChERS procedure proposal in this study clearly stands out by combining the widest analyte coverage (136 pesticides), with recoveries within the 70–120% range and LOQs of 0.01 mg kg^−1^, which are comparable to or better than those obtained with the literature methods. In addition, the present method is the only one among the approaches summarized in Table 2 that systematically includes and validates a dedicated set of twelve acidic pesticides, a group of analytes that is particularly challenging in highly acidic citrus matrices and is not adequately addressed by conventional acetate-buffered QuEChERS protocols. This expanded and more realistic coverage of both conventional and acidic pesticides, together with acceptable matrix-matched quantification performance, demonstrates that the developed method offers a suitable tool for routine residue monitoring in Tahiti lime.

### 3.4. Evaluation of Commercial Samples

Analysis of 22 Tahiti lime (*Citrus latifolia*) commercial samples using the validated UHPLC–MS/MS method (LOQ 0.01 mg kg^−1^) revealed 10 positive samples with 14 pesticides (Table 3).

Non-compliant pesticides based on the European Union MRLs were limited to thiamethoxam and dimethoate. Thiamethoxam concentrations (0.01–0.03 mg kg^−1^ in samples A3, A5 and A10) were equal to or exceeded the EU MRL (0.01 mg kg^−1^) [5] but complied with the Brazilian MRL (1.00 mg kg^−1^) [6]. Thiamethoxam is a widely used insecticide in Brazil (approximately 3300 tons in 2024) and belongs to the neonicotinoid class [32]. It is primarily applied to control a broad spectrum of sucking and chewing insect pests, including aphids, whiteflies, leafhoppers, and certain beetles. Although it remains authorized for use in several crops, its application has been increasingly scrutinized because of its potential environmental impacts, particularly on pollinators such as bees. Likewise, dimethoate concentrations (0.02–0.09 mg kg^−1^ in samples A2 and A10) exceeded the EU MRL (0.01 mg kg^−1^) [5] while remaining below the Brazilian MRL (2.00 mg kg^−1^) [6]. Dimethoate is also widely used in Brazil (approximately 800 tons in 2024) [32] and continues to play an important role in citrus production as a broad-spectrum organophosphate insecticide/acaricide for the control of economically important pests, including fruit flies (*Anastrepha* spp.) and mites. These findings illustrate the important differences between Brazilian and European regulatory frameworks and reinforce the need for analytical methods capable of supporting both domestic monitoring and export requirements. Although Tahiti lemon is not currently included in the Brazilian Pesticide Residue Analysis Program (PARA) coordinated by ANVISA [39], orange is the citrus commodity routinely monitored by the program, making official information on pesticide residues in lemons still scarce. In the 2024 monitoring cycle, ANVISA analyzed 240 orange samples, of which 93 (38.8%) were classified as unsatisfactory due to residues above the Brazilian MRLs, the presence of non-authorized pesticides, or both. Overall, 66 active ingredients were detected among the 244 pesticides investigated. Azoxystrobin (144 samples), tebuconazole (131 samples), and pyraclostrobin (94 samples) were the most frequently detected pesticides, and all three compounds are included in the analytical scope of the proposed method and were also detected in the lemon samples analyzed in the present study. Furthermore, the PARA report documented an increase in the occurrence of non-authorized pesticides in citrus, particularly profenofos, which was detected in 46 orange samples. These results further demonstrate the importance of broad-scope multiresidue analytical methods capable of simultaneously monitoring authorized and non-authorized pesticides in citrus fruits. From an international perspective, compliance with European Union legislation is particularly important for citrus exports. Consistent with the present findings, the Rapid Alert System for Food and Feed (RASFF) [40] recorded 26 notifications involving lemon consignments between 2021 and 2026, of which 22 were associated with products originating from Brazil, mainly due to pesticide residue non-compliance. Together, these findings emphasize the importance of continuous residue monitoring, implementation of good agricultural practices, and the use of comprehensive analytical methods to ensure consumer safety and compliance with international regulatory requirements.

## 4. Conclusions

A simplified and robust analytical method based on acidified acetonitrile extraction and UHPLC–MS/MS was successfully developed and validated for the multiresidue determination of 136 pesticides in Tahiti lime (*Citrus latifolia*). The use of acidified acetonitrile (1% formic acid) enabled reliable determination of acidic pesticides while eliminating the need for a clean-up step, resulting in a simple and high-throughput sample preparation procedure. The method fulfilled all validation criteria, demonstrating adequate linearity, accuracy, precision, and sensitivity for routine residue analysis. Application to commercial samples confirmed its suitability for monitoring pesticide residues in Tahiti lime and revealed residues of several pesticides, with thiamethoxam and dimethoate exceeding the European Union MRLs while remaining compliant with Brazilian legislation. Therefore, the proposed method represents a reliable and practical tool for routine multiresidue analysis, supporting food safety monitoring and regulatory compliance in citrus fruits.

## Figures and Tables

**Table 1:** Summary of the validation performance of the proposed UHPLC–MS/MS method for the determination of 136 pesticides in Tahiti lime.

Validation Parameter	Summary of Results
Number of validated analytes	136 pesticides
Selectivity	No interfering peaks were observed at the retention times of the target analytes in blank samples.
Linearity	R^2^ = 0.9900–1.0000
Recovery	70–120% at the three fortification levels (0.01, 0.025 and 0.05 mg kg^−1^)
Repeatability	RSD ≤ 20%
Within-laboratory reproducibility	RSD ≤ 20%
Matrix effect (ME)	48.5% signal suppression, 48.5% negligible ME, 3% signal enhancement
LOQ	0.01 mg kg^−1^ for all analytes

LOQ: limit of quantification; R^2^: coefficient of determination; RSD: relative standard deviation.

**Table 2:** Comparative overview of multiresidue sample preparation methods for pesticide analysis in citrus matrices.

Sample Preparation	Instrumentation	No. of Pesticides	Recovery (%)	Matrix Effect	LOQ (mg kg^−1^)	Acidic Pesticides Included	Ref.
Magnetic solid-phase extraction	LC–MS/MS	20	74.3–92.3	Matrix-matched calibration	0.001	No	[36]
QuEChERS	LC–MS/MS	74	70–118	Matrix-matched calibration	0.01	No	[37]
QuEChERS	LC–MS/MS	115	81.7–111	Not evaluated	0.01	Yes (3 compounds)	[38]
QuEChERS acidified	UHPLC–MS/MS	136	70–120	Matrix-matched calibration	0.01	Yes (12 compounds)	[This study]

**Table 3:** Pesticide residues detected by UHPLC–MS/MS in positive Tahiti lime (*Citrus latifolia*) samples: LOQ concentrations (mg kg^−1^), Brazil and European Union (EU) Maximum Residue Limits (MRLs) (mg kg^−1^) [5,6] across 10 samples.

Pesticides	LOQ (mg/kg)	Brazil (MRL)	EU (MRL)	A3	A5	A9	A10	A11	A12	A13	A14	A15	A19
Azoxystrobin	all 0.01	7.00	15.0	nd	nd	nd	nd	<LOQ	nd	0.01	nd	nd	nd
Carbendazim		-	0.70	nd	nd	nd	nd	nd	0.04	nd	nd	nd	nd
Clothianidin		0.3	0.01	nd	<LOQ	nd	nd	nd	nd	nd	nd	nd	nd
Diazinon		-	0.01	nd	nd	nd	nd	nd	nd	nd	<LOQ	nd	nd
Difenoconazole		0.5	0.60	nd	nd	nd	nd	nd	nd	<LOQ	nd	nd	nd
Dimethoate		2.00	0.01	nd	0.02	nd	0.09	nd	nd	nd	nd	nd	nd
Imazalil		5.00	5.00	nd	nd	nd	nd	0.5	1.73	1.00	2.65	0.01	nd
Imidacloprid		2.00	0.90	nd	0.06	nd	0.05	nd	nd	nd	0.01	nd	0.04
Metalaxyl		2.00	0.40	nd	nd	nd	nd	nd	0.03	nd	nd	nd	nd
Pyraclostrobin		0.50	2.00	nd	<LOQ	nd	<LOQ	nd	nd	nd	nd	nd	<LOQ
Pyrimethanil		5.00	8.00	nd	nd	nd	nd	nd	1.30	0.01	1.64	<LOQ	nd
Tebuconazole		5.00	5.00	nd	0.05	0.01	0.04	nd	nd	0.03	nd	nd	nd
Thiabendazole		10.00	7.00	nd	nd	nd	nd	nd	0.04	nd	0.64	nd	nd
Thiamethoxam		1.00	0.01	0.01	0.03	nd	0.03	nd	nd	nd	nd	nd	nd

LOQ: limit of quantification; MRL: maximum residue limit; nd: not detected; <LOQ: lower than limit of quantification.

## Data Availability

The original contributions presented in this study are included in the article/Supplementary Materials. Further inquiries can be directed to the corresponding author.

## Supplementary Materials

The following supporting information can be downloaded at https://www.mdpi.com/article/10.3390/foods15172967/s1; Table S1. Pesticides analyzed by UHPLC–MS/MS system with their respective retention times (tR, min), MRM transitions, and fragmentation energies (eV) used. Table S2. UHPLC–MS/MS validation parameters for 136 pesticides: linearity (R^2^), recovery (%), repeatability (%RSD, *n* = 6), and within-laboratory reproducibility (%RSD, *n* = 6) across different concentrations (0.01, 0.025, 0.05 mg kg^−1^).

## Abbreviations

The following abbreviations are used in this manuscript:

ANVISA	Brazilian Health Regulatory Agency/Agência Nacional de Vigilância Sanitária
dSPE	dispersive Solid-Phase Extraction
ESI−	Negative Electrospray Ionization
ESI+	Positive Electrospray Ionization
EU	European Union
EURL-SRM	European Union Reference Laboratory for Single Residue Methods
GAPs	Good Agricultural Practices
LOD	Limit of Detection
LOQ	Limit of Quantification
ME	Matrix Effect
MgSO_4_	Magnesium Sulfate
MRLs	Maximum Residue Limits
MRM	Multiple Reaction Monitoring
MS/MS	Tandem Mass Spectrometry
NaCl	Sodium Chloride
nd	Not detected
PSA	Primary Secondary Amine
PVDF	Polyvinylidene Fluoride
QuEChERS	Quick, Easy, Cheap, Effective, Rugged, and Safe
RSD	Relative Standard Deviation
S/N	Signal-to-Noise Ratio
SANTE	Health and Food Safety Directorate-General guidance document
UHPLC	Ultra-High Performance Liquid Chromatography
UHPLC–MS/MS	Ultra-High Performance Liquid Chromatography coupled with Tandem Mass Spectrometry
