# Supplementary material for: Acidified Acetonitrile Extraction and Ultra-High Performance Liquid Chromatography–Tandem Mass Spectrometry for Multiresidue Determination of 136 Pesticides in Tahiti Lime (Citrus latifolia)
Source: Foods. 2026 Aug 24;15(17):2967. doi: 10.3390/foods15172967 (PMC13565522; doi:10.3390/foods15172967)
Supplement: Supplementary file 1 [file foods-15-02967-s001.zip › foods-4362406-supplementary.pdf]

Acidified Acetonitrile Extraction and Ultra-High Performance Liquid Chromatography–Tandem Mass Spectrometry for Multiresidue Determination of 136 Pesticides in Tahiti Lime (*Citrus latifolia*)

Karolaine da Silva Albarnaz, Juliana Diniz de Barros Kuntz, Luana Floriano, Franciele Fátima Machado, Martha Bohrer Adaime, Renato Zanella and Osmar Damian Prestes *

Laboratory of Pesticide Residue Analysis (LARP), Department of Chemistry, Federal University of Santa Maria, Av. Roraima, 1000, Santa Maria 97105-900, RS, Brazil
* Correspondence: osmar.prestes@ufsm.br; Tel.: +55-553220-8011

Table S1. Pesticides analyzed by UHPLC–MS/MS system with their respective retention times (tR, min), MRM transitions, and fragmentation energies (eV) used.

	Analytes	Cone voltage (V)	Precursor ion (m/z)	1st transition (quantification)		2nd transition (quantification)	
				Product ion (m/z)	Cone energy (eV)	Product ion (m/z)	Cone energy (eV)
1	2,4,5-T	15	253.0	195.0	18	197.0	18
2	2,4,6-TCP	40	195.0	35.0	20	159.0	20
3	2,4 D	12	219.0	124.9	26	160.9	10
4	Acetamiprid	23	223.0	126.0	20	56.1	15
5	Alachlor	20	270.1	238.1	11	162.2	20
6	Ametrine	32	228.1	186.1	18	68.1	36
7	Atrazine	30	216.2	174.1	18	96.1	23
8	Azaconazole	25	300.0	159.0	28	231.1	18
9	Azamethiphos	31	325.0	111.9	35	138.9	24
10	Azoxystrobin	17	404.1	372.0	30	328.9	15
11	Benalaxyl	17	326.1	148.0	20	91.0	34
12	Boscalida	32	342.9	139.9	20	307.0	20
13	Bromacil	30	259.0	203.0	18	204.9	14
14	Bromoconazole	32	376.0	70.1	25	158.9	35
15	Buprofezin	22	306.1	201.0	12	115.9	20
16	Carbendazim	24	192.1	132.1	30	160.1	18
17	Carbofuran	25	222.1	165.1	16	123.0	16
18	Chlorantraniliprole	20	481.6	283.9	22	451.1	14
19	Chlorimuron-ethyl	25	415.1	186.0	17	83.0	15
20	Chlorthiophos	28	360.9	154.9	12	98.9	30
21	Clomazone	23	240.0	125.0	46	89.0	18
22	Clothianidin	15	250.0	169.0	18	132.0	12
23	Cyanazine	28	241.0	214.0	17	96.0	25
24	Cyazofamid	17	325.0	107.9	20	261.0	10
25	Cymoxanil	15	199.2	128.2	10	111.1	20
26	Cyproconazole	27	292.2	70.2	24	125.1	18
27	Cyprodinil	46	226.0	93.0	25	108.0	33
28	Demeton-S-methyl-sulfone	20	263.0	169.0	17	121.0	17
29	Diazinon	20	305.1	169.0	35	96.9	22
30	Dichlofluanid	19	333.0	123.0	35	224.0	25

	Analytes	Cone voltage (V)	Precursor ion (m/z)	1st transition (quantification)		2nd transition (quantification)	
				Product ion (m/z)	Cone energy (eV)	Product ion (m/z)	Cone energy (eV)
31	Diclosulam	46	406.1	161.0	30	378.0	15
32	Difenoconazole	37	406.0	251.1	25	111.1	60
33	Diflubenzuron	34	311.1	158.2	12	141.1	28
34	Dimethoate	12	230.0	198.8	20	124.8	10
35	Dimethomorph	30	388.1	165.0	30	300.9	20
36	Diniconazole	37	326.1	70.2	25	159.0	34
37	Diurom	27	233.0	72.0	14	159.9	18
38	Epoxiconazole	25	330.0	121.0	50	101.0	22
39	Ethiofencarb sulfoxide	19	242.1	185.1	18	107.1	8
40	Ethoprophos	18	243.0	131.0	31	97.0	20
41	Ethoxysulfuron	25	398.9	261.0	16	218.0	24
42	Fembuconazole	29	337.0	125.0	3	70.1	40
43	Fempropimorph	41	304.2	147.1	28	57.2	30
44	Fenamidone	22	312.1	92.0	14	236.1	25
45	Fenarimol	17	331.0	81.0	34	268.0	22
46	Fenhexamide	32	302.0	97.1	22	55.2	38
47	Flonicamid	35	230.1	203.1	15	203.1	18
48	Flupicolide	40	383.0	172.9	15	365.0	20
49	Fluquinconazole	37	376.0	348.4	18	306.9	30
50	Fluroxypyr	28	254.9	180.8	22	208.8	16
51	Flusilazole	27	316.0	165.0	28	247.0	18
52	Flutolanil	23	324.1	262.1	40	65.0	18
53	Flutriafol	23	302.1	70.1	29	122.9	18
54	Imazalil	31	297.1	69.1	18	159.0	22
55	Imazamox	25	306.1	218.0	30	278.0	20
56	Imazapique	35	276.2	231.2	20	163.0	26
57	Imazapyr	38	262.2	69.2	26	86.1	26
58	Imazaquim	40	312.2	267.2	20	86.2	28
59	Imazethapyr	45	290.2	177.1	25	245.2	20
60	Imidacloprid	23	256.1	209.0	20	174.9	15
61	Iprodione	36	330.2	245.1	15	288.1	5
62	Iprovalicarb	20	321.1	119.1	20	203.1	10
63	Linurom	25	249.0	159.9	16	181.9	18
64	Malathion	12	331.0	126.9	24	98.9	12
65	MCPA	30	199.0	141.0	13	140.9	15
66	Mecarbam	12	330.0	227.1	35	97.0	8
67	Mepanipyrim	15	224.1	77.0	35	106.0	25
68	Mephospholan	25	270.1	140.0	25	75.1	15
69	Mepronil	27	270.1	119.0	44	91.0	28
70	Metalaxyl	15	280.1	220.1	17	192.1	13
71	Metconazole	29	320.1	70.0	22	125.0	36
72	Methamidophos	30	141.9	93.9	12	124.8	14
73	Methiocarb	19	226.0	169.0	22	121.0	10
74	Methiocarb-sulfone	31	258.1	107.1	38	122.1	19
75	Methiocarb-sulfoxide	17	242.0	185.0	28	122.0	14
76	Methomyl	17	162.9	88.0	10	105.9	10

	Analytes	Cone voltage (V)	Precursor ion (m/z)	1st transition (quantification)		2nd transition (quantification)	
				Product ion (m/z)	Cone energy (eV)	Product ion (m/z)	Cone energy (eV)
77	Metolachlor	17	284.1	252.1	25	176.1	15
78	Metsulfuron-methyl	22	382.0	167.0	22	198.9	16
79	Molinato	17	188.0	126.0	13	55.0	27
80	Monocrotophos	15	224.1	127.1	12	98.1	16
81	Monolinuron	23	215.0	126.0	34	99.0	22
82	Myclobutanil	25	289.1	70.2	18	125.1	32
83	Nicosulfuron	26	411.0	182.0	22	106.0	32
84	Nuarimol	37	315.0	252.0	22	81.1	28
85	Omethoate	16	214.0	182.8	22	124.8	11
86	Oxadixyl	20	279.1	219.0	12	132.3	25
87	Paraoxon-ethyl	28	276.1	220.1	17	248.2	16
88	Parathion-methyl	29	263.9	109.0	22	79.0	36
89	Pencicuron	20	329.1	124.9	24	218.0	60
90	Penconazole	25	284.0	70.1	34	159.0	16
91	Penoxsulam	42	484.2	195.1	30	164.1	40
92	Phorato	15	261.0	75.0	12	199.0	5
93	Phosmet	19	318.0	160.0	22	77.0	46
94	Picoxystrobin	10	368.0	145.1	22	205.1	10
95	Piperonyl butoxide	20	356.3	176.9	10	119.0	35
96	Pirimicarb	25	239.1	72.4	15	182.1	18
97	Pirimiphos-ethyl	30	334.1	198.1	23	182.1	25
98	Pirimiphos-methyl	25	306.3	164.1	32	108.1	22
99	Prochloraz	10	375.8	70.1	24	265.8	16
100	Procymidone	33	368.0	145.0	17	205.0	28
101	Profenofos	25	372.9	302.6	40	127.9	20
102	Prometryn	26	242.0	158.0	25	200.1	17
103	Propanil	31	217.9	161.9	16	127.0	22
104	Propiconazole	37	342.1	158.9	34	69.1	22
105	Propyzamide	31	256.1	190.0	16	173.0	23
106	Pyraclostrobin	20	388.1	163.0	12	193.9	25
107	Pyrazophos	33	374.0	222.1	32	194.0	22
108	Pyrazosulfuron-ethyl	22	415.0	182.0	20	82.9	45
109	Pyridaphenthion	31	341.0	189.0	34	92.0	22
110	Pyrimethanil	42	200.0	107.0	24	82.0	24
111	Quinalphos	15	299.0	162.9	30	96.9	24
112	Quinoxyfen	52	308.0	197.0	44	161.9	33
113	Quizalofop-ethyl	30	373.0	91.1	32	299.1	18
114	Quizalofop-ethyl	25	373.0	299.1	24	91.1	36
115	Saflufenacil	30	501.1	198.0	30	349.0	49
116	Simazine	34	202.0	124.0	22	96.0	16
117	Spinosad A	47	732.6	142.0	59	98.1	31
118	Spinosad D	42	746.5	142.0	53	98.1	31
119	Tebuconazole	34	308.2	70.1	22	124.9	16
120	Tebufempirade	43	334.0	145.0	34	117.0	28
121	Tetraconazole	15	372.0	159.0	25	70.1	20
122	Thiabendazole	45	202.0	130.9	30	174.9	25

	Analytes	Cone voltage (V)	Precursor ion (m/z)	1st transition (quantification)		2nd transition (quantification)	
				Product ion (m/z)	Cone energy (eV)	Product ion (m/z)	Cone energy (eV)
123	Thiacloprid	32	253.0	125.8	20	90.0	40
124	Thiamethoxam	19	292.0	132.0	22	211.2	12
125	Thiobencarb	21	258.1	125.1	17	89.1	12
126	Thiodicarb	17	355.1	88.1	16	108.1	16
127	Triadimefon	30	294.1	69.1	20	196.9	16
128	Triadimenol	30	296.1	70.0	10	98.9	15
129	Trichlorfon	25	256.9	108.8	20	126.8	18
130	Triclopyr	20	254.0	196.0	12	198.0	12
131	Tricyclazole	10	190.0	163.0	20	136.0	25
132	Trifloxysulfuron	30	438.0	182.0	20	139.0	45
133	Triflumizole	13	346.1	278.0	10	73.1	10
134	Triflumuron	23	359.0	156.1	35	139.1	16
135	Vamidathion	17	288.0	146.0	10	118.0	28
136	Zoxamide	27	336.0	187.1	25	159.0	38
	Atrazine-d5 (PC)	26	221.1	179.3	18	101.0	23
	Triphenylphosphate (PI)	40	327.2	152.1	37	215.0	28

Table S2. UHPLC–MS/MS validation parameters for 136 pesticides: linearity (R^2), recovery (%), repeatability (%RSD, n=6), and within-laboratory reproducibility (%RSD, n=6) across different concentrations (0.01, 0.025, 0.05 mg kg^{-1}).

nº	analites	R^2	Recovery (%) Repeatability (%RSD, n=6)			Within-laboratory reproducibility (%RSD, n=6)		
			0.01	0.025	0.05	0.01	0.025	0.05
1	2,4,5-T	0.9993	77(11)	74(17)	76(6)	80(6)	92(8)	83(13)
2	2,4,6-TCP	0.9995	88(5)	92(5)	87(4)	79(3)	82(3)	76(3)
3	2,4-D	0.9997	118(6)	119(2)	113(3)	86(6)	87(8)	82(8)
4	Acetamiprid	0.9978	116(11)	116(4)	109(7)	109(6)	100(16)	92(7)
5	Alachlor	0.9999	100(5)	100(6)	94(4)	95(7)	92(12)	85(4)
6	Ametryn	0.9997	91(4)	96(2)	91(3)	97(10)	93(6)	87(3)
7	Atrazine	0.9994	77(10)	104(17)	96(6)	96(10)	98(13)	88(6)
8	Azaconazole	0.9996	101(5)	102(2)	93(6)	98(8)	97(7)	91(6)
9	Azamethiphos	0.9993	104(6)	97(6)	91(4)	103(9)	98(10)	93(4)
10	Azoxystrobin	0.9999	93(5)	92(3)	89(2)	103(8)	103(6)	91(2)
11	Benalaxyl	0.9960	94(6)	98(8)	90(4)	95(14)	96(13)	82(4)
12	Boscalid	0.9998	111(8)	103(9)	96(4)	98(7)	98(7)	87(4)
13	Bromacil	0.9999	78(10)	71(10)	76(9)	72(20)	87(13)	82(15)
14	Bromuconazole	0.9999	95(5)	95(8)	97(7)	88(6)	96(6)	91(7)
15	Buprofezin	0.9999	92(4)	92(3)	89(4)	104(8)	100(6)	91(4)
16	Carbendazim	0.9998	96(3)	99(4)	97(4)	84(9)	90(4)	80(4)
17	Carbofuran	0.9986	111(4)	110(03)	104(3)	110(8)	104(5)	92(3)
18	Chlorantraniliprole	0.9998	91(10)	86(8)	87(6)	119(9)	105(14)	100(6)
19	Chlorimuron-ethyl	0.9998	94(6)	90(5)	87(3)	98(10)	98(9)	88(3)
20	Chlorthiophos	0.9996	102(5)	94(9)	87(5)	97(11)	93(17)	83(5)
21	Clomazone	0.9993	101(11)	99(2)	93(3)	104(7)	93(11)	89(3)
22	Clothianidin	0.9993	114(4)	117(6)	107(5)	114(11)	105(13)	92(5)
23	Cyanazine	0.9981	112(6)	104(7)	88(6)	117(7)	116(9)	99(6)

nº	analites	R^2	Recovery (%) Repeatability (%RSD, n=6)			Within-laboratory reproducibility (%RSD, n=6)		
			0.01	0.025	0.05	0.01	0.025	0.05
24	Cyazofamid	0.9901	92(17)	91(17)	75(13)	92(13)	97(6)	82(13)
25	Cymoxanil	0.9987	94(4)	112(4)	95(3)	71(13)	86(7)	81(5)
26	Cyproconazole	0.9998	96(4)	92(3)	87(4)	94(12)	99(9)	92(4)
27	Cyprodinil	0.9999	88(7)	93(10)	89(6)	93(12)	97(5)	93(6)
28	Demeton-S-methyl sulfone	1.0000	116(4)	115(10)	118(7)	98(17)	106(15)	91(7)
29	Diazinon	0.9981	81(5)	70(4)	72(5)	85(10)	73(16)	80(5)
30	Dichlofluanid	0.9959	94(7)	87(12)	91(8)	89(8)	97(18)	92(8)
31	Diclosulam	0.9979	95(6)	100(1)	96(3)	99(14)	105(8)	98(3)
32	Difenoconazole	0.9968	111(8)	98(17)	87(6)	97(14)	99(14)	76(6)
33	Diflubenzuron	0.9985	104(6)	96(7)	87(4)	98(11)	93(14)	82(4)
34	Dimethoate	0.9992	119(3)	117(5)	103(8)	112(6)	105(8)	92(8)
35	Dimethomorph	0.9996	89(6)	102(3)	96(2)	76(7)	85(4)	79(5)
36	Diniconazole	0.9997	104(6)	97(6)	93(5)	95(8)	90(12)	82(5)
37	Diuron	0.9996	102(5)	103(5)	98(5)	105(8)	96(8)	91(5)
38	Epoxiconazole	0.9998	94(9)	94(7)	90(5)	101(9)	95(7)	87(5)
39	Ethiofencarb sulfoxide	0.9910	114(4)	112(3)	98(9)	114(15)	115(14)	98(9)
40	Ethoprophos	0.9998	74(7)	90(1)	89(4)	106(12)	97(8)	87(4)
41	Ethoxysulfuron	0.9992	86(6)	90(2)	87(4)	95(9)	94(8)	86(4)
42	Fembuconazole	0.9995	89(5)	94(9)	91(4)	94(12)	99(11)	89(4)
43	Fenpropimorph	0.9997	101(6)	98(2)	96(4)	101(7)	102(3)	90(4)
44	Fenamidone	0.9997	91(5)	90(1)	833)	101(9)	95(9)	85(3)
45	Fenarimol	0.9999	96(6)	92(5)	87(4)	108(9)	100(8)	89(4)
46	Fenhexamid	1.0000	111(11)	104(8)	96(7)	97(9)	96(10)	89(7)
47	Flonicamid	1.0000	97(7)	108(4)	103(5)	75(4)	79(1)	77(9)
48	Fluopicolide	0.9996	91(7)	98(10)	95(3)	93(11)	91(14)	84(3)
49	Fluquinconazole	0.9998	114(6)	108(14)	99(5)	92(10)	93(9)	87(5)
50	Fluroxypyr	0.9995	113(4)	119(3)	110(4)	73(4)	82(7)	78(9)
51	Flusilazole	0.9992	97(8)	92(6)	87(5)	101(11)	99(14)	91(5)
52	Flutolanil	0.9993	104(4)	97(6)	91(2)	98(9)	96(9)	86(2)
53	Flutriafol	0.9997	101(5)	100(3)	94(4)	99(8)	95(8)	86(4)
54	Imazalil	0.9999	87(4)	93(4)	95(4)	87(6)	94(3)	87(1)
55	Imazamox	0.9995	110(7)	99(3)	92(7)	72(8)	73(11)	71(3)
56	Imazapic	0.9994	100(9)	103(5)	102(8)	102(12)	101(13)	96(8)
57	Imazapyr	0.9997	90(10)	94(7)	96(3)	77(6)	81(5)	75(4)
58	Imazaquin	0.9994	95(7)	90(2)	83(7)	110(9)	104(9)	94(7)
59	Imazethapyr	0.9998	85(5)	93(5)	87(5)	74(4)	81(5)	77(4)
60	Imidacloprid	0.9997	108(14)	108(4)	103(5)	99(7)	94(10)	89(5)
61	Iprodione	0.9975	109(5)	100(7)	91(5)	93(13)	92(18)	84(5)
62	Iprovalicarb	0.9999	97(4)	104(2)	98(3)	77(5)	77(5)	73(7)
63	Linuron	0.9982	94(8)	93(5)	92(4)	93(9)	100(6)	92(4)
64	Malathion	0.9900	99(14)	100(19)	84(12)	98(19)	83(20)	72(12)
65	MCPA	0.9981	81(8)	81(12)	76(6)	92(8)	95(5)	94(4)
66	Mecarbam	0.9994	92(9)	89(13)	83(5)	95(16)	93(13)	79(5)
67	Mepanipyrim	0.9994	88(11)	94(5)	86(2)	103(13)	94(11)	85(2)
68	Mephospholan	0.9990	99(9)	96(4)	88(6)	111(9)	108(11)	98(6)
69	Mepronil	0.9997	95(5)	91(3)	89(2)	98(7)	95(9)	86(2)
70	Metalaxyl	0.9998	93(4)	99(5)	93(8)	104(8)	98(9)	96(8)
71	Metconazole	0.9998	105(5)	99(7)	98(4)	95(9)	95(14)	89(4)

nº	analites	R^2	Recovery (%) Repeatability (%RSD, n=6)			Within-laboratory reproducibility (%RSD, n=6)		
			0.01	0.025	0.05	0.01	0.025	0.05
72	Methamidophos	0.9997	89(4)	89(8)	80(5)	71(1)	72(6)	78(4)
73	Methiocarb	0.9992	98(12)	95(4)	87(4)	104(7)	99(7)	86(4)
74	Methiocarb sulfone	0.9999	91(4)	101(8)	101(3)	70(2)	76(5)	70(4)
75	Methiocarb sulfoxide	0.9981	117(3)	120(5)	117(4)	118(16)	114(14)	98(4)
76	Methomyl	0.9993	116(3)	116(4)	114(5)	102(11)	95(14)	89(5)
77	Metolachlor	0.9998	91(6)	101(6)	94(2)	93(12)	98(8)	89(2)
78	Metsulfuron-methyl	0.9995	95(7)	95(4)	93(4)	109(8)	95(11)	88(4)
79	Molinate	0.9991	95(6)	95(7)	88(9)	92(15)	98(10)	92(9)
80	Monocrotophos	0.9988	117(11)	118(3)	112(7)	95(8)	96(6)	89(7)
81	Monolinuron	0.9997	111(6)	102(6)	96(5)	91(6)	87(5)	85(5)
82	Myclobutanil	0.9987	91(5)	96(3)	94(3)	107(7)	99(9)	88(3)
83	Nicosulfuron	0.9984	107(6)	97(6)	88(5)	109(10)	104(9)	98(5)
84	Nuarimol	0.9997	98(5)	97(2)	92(5)	99(9)	96(9)	89(5)
85	Omethoate	0.9962	115(5)	100(5)	91(6)	93(5)	89(13)	81(6)
86	Oxadixyl	0.9992	90(13)	93(8)	84(3)	110(4)	115(6)	100(3)
87	Paraoxon-ethyl	0.9994	101(5)	105(2)	95(8)	96(11)	94(5)	86(8)
88	Parathion-methyl	0.9993	118(8)	111(4)	114(13)	109(19)	108(17)	90(13)
89	Pencycuron	0.9905	104(12)	94(17)	82(9)	92(15)	101(14)	76(9)
90	Penconazole	0.9981	103(7)	97(6)	92(4)	94(7)	93(13)	85(4)
91	Penoxsulam	0.9999	96(5)	97(3)	90(9)	107(9)	96(7)	91(9)
92	Phorate	0.9993	112(20)	106(16)	96(13)	101(13)	83(17)	75(13)
93	Phosmet	0.9996	105(10)	99(6)	89(4)	97(13)	91(10)	87(4)
94	Picoxystrobin	0.9912	71(11)	83(20)	82(12)	106(16)	83(20)	70(12)
95	Piperonyl butoxide	0.9966	93(10)	81(13)	85(14)	94(13)	86(13)	72(14)
96	Pirimicarb	0.9993	88(12)	87(9)	82(8)	102(6)	107(7)	82(8)
97	Pirimiphos-ethyl	0.9996	98(4)	92(9)	87(2)	100(10)	87(17)	77(2)
98	Pirimiphos-methyl	0.9998	90(6)	88(4)	85(3)	104(9)	94(10)	86(3)
99	Prochloraz	1.0000	95(5)	98(6)	95(9)	90(7)	90(7)	82(6)
100	Procymidone	0.9900	103(19)	74(2)	96(16)	109(20)	100(12)	79(16)
101	Profenofos	0.9928	96(10)	81(8)	80(12)	100(12)	101(13)	70(12)
102	Prometryn	0.9988	87(6)	90(2)	87(5)	102(9)	99(6)	91(5)
103	Propanil	0.9999	101(3)	96(4)	93(4)	98(15)	95(5)	89(4)
104	Propiconazole	0.9998	99(11)	102(8)	95(4)	93(8)	89(12)	82(4)
105	Propyzamide	0.9997	98(6)	94(6)	87(10)	98(6)	94(7)	85(10)
106	Pyraclostrobin	0.9900	85(13)	82(12)	90(12)	100(11)	87(18)	80(12)
107	Pyrazophos	0.9926	102(12)	90(17)	81(9)	98(17)	92(17)	71(9)
108	Pyrazosulfuron-ethyl	0.9999	93(5)	92(1)	87(3)	103(10)	97(8)	90(3)
109	Pyridaphenthion	0.9997	98(7)	100(5)	92(8)	98(12)	100(9)	92(8)
110	Pyrimethanil	0.9998	89(7)	95(1)	92(5)	90(11)	95(6)	88(5)
111	Quinalphos	0.9982	102(4)	93(8)	83(6)	97(13)	87(20)	76(6)
112	Quinoxyfen	0.9992	103(8)	90(17)	80(9)	97(12)	90(14)	88(9)
113	Quizalofop-ethyl	0.9903	78(12)	73(14)	88(11)	93(12)	91(17)	75(11)
114	Rotenone	0.9999	114(8)	97(6)	98(7)	92(17)	97(14)	77(7)
115	Saflufenacil	0.9999	98(6)	96(7)	93(4)	105(11)	1028)	93(4)
116	Simazine	0.9991	99(8)	97(5)	88(10)	119(6)	108(7)	99(10)
117	Spinosad A	0.9998	111(3)	116(3)	120(6)	112(10)	118(7)	101(6)
118	Spinosad D	0.9995	116(3)	117(3)	113(6)	119(3)	118(5)	113(6)
119	Tebuconazole	0.9997	96(8)	91(3)	87(3)	96(7)	93(9)	86(3)

nº	analites	R^2	Recovery (%) Repeatability (%RSD, n=6)			Within-laboratory reproducibility (%RSD, n=6)		
			0.01	0.025	0.05	0.01	0.025	0.05
120	Tebufenpyrad	0.9906	88(11)	87(12)	80(14)	102(13)	94(17)	75(14)
121	Tetraconazole	0.9986	99(6)	94(7)	90(5)	99(8)	95(11)	87(9)
122	Thiabendazole	0.9997	87(4)	95(6)	94(5)	75(7)	77(6)	71(9)
123	Thiacloprid	0.9991	113(8)	99(6)	92(6)	118(12)	105(13)	90(6)
124	Thiamethoxam	1.0000	112(14)	116(9)	109(9)	106(15)	101(17)	89(9)
125	Thiobencarb	0.9944	97(6)	88(13)	79(6)	96(14)	93(10)	78(6)
126	Thiodicarb	1.0000	93(4)	100(3)	98(5)	73(8)	72(3)	74(5)
127	Triadimefon	0.9996	97(6)	93(3)	89(3)	98(9)	96(8)	89(3)
128	Triadimenol	0.9918	82(12)	92(8)	93(7)	106(10)	99(9)	90(7)
129	Trichlorfon	0.9984	115(3)	113(8)	98(10)	108(12)	106(9)	95(10)
130	Triclopyr	0.9999	99(12)	102(4)	97(4)	93(17)	113(18)	113(9)
131	Tricyclazole	0.9980	105(1)	120(6)	110(11)	117(14)	104(14)	95(11)
132	Trifloxysulfuron	0.9997	102(6)	102(2)	91(11)	92(10)	97(7)	92(11)
133	Triflumizole	0.9999	88(6)	83(7)	84(8)	97(7)	91(11)	80(8)
134	Triflumuron	0.9905	98(16)	89(15)	76(13)	93(19)	91(16)	71(13)
135	Vamidothion	0.9986	120(10)	111(5)	97(6)	112(15)	106(14)	956)
136	Zoxamide	0.9967	96(7)	97(11)	87(4)	96(14)	95(13)	76(4)
